# Comparison of *In Vitro* Biofilm Formation on Titanium and Zirconia Implants

**DOI:** 10.1155/2023/8728499

**Published:** 2023-04-15

**Authors:** Lan-Lin Chiou, Beatriz H. D. Panariello, Yusuke Hamada, Richard L. Gregory, Steven Blanchard, Simone Duarte

**Affiliations:** ^1^Department of Periodontology, Indiana University School of Dentistry, Indianapolis, IN, USA; ^2^Department of Biomedical Sciences and Comprehensive Care, Indiana University School of Dentistry, Indianapolis, IN, USA; ^3^American Dental Association Science and Research Institute, Chicago, IL, USA

## Abstract

**Background:**

Peri-implant diseases are emerging issues in contemporary implant dentistry. As biofilms play a critical role in peri-implant diseases, the characteristic of resisting bacterial adhesion would be ideal for dental implants. The aims of the study were to compare titanium (Ti) and zirconia (Zr) implants regarding the amount of biofilm formation at different time frames and assess the distribution of biofilm on different aspects of dental implants.

**Methods:**

Biofilm was developed on Ti and Zr dental implants with a peri-implant-related multispecies model with *Streptococcus oralis*, *Actinomyces naeslundii*, *Veillonella dispar*, and *Porphyromonas gingivalis*, for 3 and 14 days. Quantitative assessment was performed with the measurement of total bacterial viability (colony forming units, CFU/mg). Scanning electron microscopy (SEM) was used to evaluate biofilm formation on different aspects of the implants.

**Results:**

Three-day-old biofilm on Ti implants was significantly higher than that on Zr implants (*p* < 0.001). The Ti and Zr groups were not significantly different for 14-day-old biofilm. SEM images demonstrated that 3-day-old biofilm on Zr implants was sparse while biofilm growth was more pronounced for 3-day-old biofilm on Ti implants and 14-day-old biofilm groups. It appeared that less biofilm formed on the valley compared to the thread top for 3-day-old biofilm on Zr implants. Differences between the valley and the thread top became indistinguishable with the development of mature biofilm.

**Conclusion:**

While early formed biofilms show greater accumulation on Ti implants compared to Zr implants, older biofilms between the two groups are comparable. The distribution of biofilms was not uniform on different areas of implant threads during early biofilm development.

## 1. Introduction

As oral rehabilitation with utilizing dental implants improves masticatory function, patient satisfaction, and oral health-related quality of life [1, 2], the prevalence of dental implants in the United States has substantially increased. Although the number of dental implants placed is projected to grow significantly, peri-implant diseases, inflammatory conditions around soft and/or hard tissues around dental implants, have become emerging issues in contemporary implant dentistry. A meta-analysis published in 2017 revealed that the prevalence of peri-implantitis at the subject and implant levels was 19.83% and 9.25%, respectively, while the prevalence of peri-implant mucositis at the subject and implant levels was 46.83% and 29.48%, respectively [3]. Numerous factors may contribute to the development and progression of peri-implant diseases [4]. Biofilm formation is considered to be one of the etiologic factors for peri-implant mucositis and peri-implantitis [5]. The sequence of biofilm formation around dental implants is similar to that around natural teeth. After an acquired pellicle forms on the implant surface, adhesion of early colonizing bacteria occurs, followed by late colonizers [6–8]. Furst et al., with the use of titanium transmucosal implants, found that colonization of bacteria occurred within 30 minutes after the completion of the surgical implant installation [9]. The presence of early colonizers, including *Streptococcus*, *Actinomyces*, *Capnocytophaga*, and *Veillonella* species, as well as middle and late colonizers such as *Fusobacterium nucleatum*, *Porphyromonas gingivalis*, *Treponema denticola*, and *Tannerella forsythia* in biofilms on dental implant surfaces, has been reported [7, 9, 10]. Furthermore, Kumar et al. demonstrated that Gram-negative species predominated in the peri-implant biofilms along with higher levels of *Actinomyces*, *Peptococcus*, *Campylobacter*, nonmutans *Streptococci*, *Butyrivibrio*, and *Streptococcus mutans* in the peri-implantitis sites [10].

Clinically, peri-implantitis with titanium dental implants can occur within three years of function, and the progression of peri-implantitis occurs in a nonlinear and accelerating pattern [11]. On the other hand, Roehling et al. showed that bone loss around zirconia implants was significantly less than that around titanium implants by utilizing ligature-induced inflammation in a canine model [12]. Nevertheless, regarding the long-term clinical performance, it is difficult to compare zirconia implants with titanium implants as zirconia implants are relatively new to the dental implant market. For commercially available zirconia implants, the mean 1-year marginal bone loss was 0.7 mm, and the overall incidence of biological complications, including soft tissue complications and peri-implantitis, was 4.2% on the basis of 1- to 5-year studies [13]. *In vitro* studies comparing biofilm formation on zirconia surfaces versus that on titanium surfaces yielded conflicting results, however. Using titanium and zirconium dioxide disks, some investigations exhibited reduced bacterial adhesion on the zirconia surfaces compared to that on the titanium surfaces [14–16] whereas others revealed that no statistically significant difference between zirconia and titanium surfaces could be observed [17, 18]. Various study designs may account for the heterogeneity of the results.

The physicochemical characteristics of implant surfaces have long been discussed as factors influencing bacterial attachment and biofilm formation on the implant surfaces. Generally, increased bacterial adhesion is positively correlated with rough or hydrophobic surfaces, indicating that materials and surface treatments could influence the biofilm formation on dental implants [19–21]. With the use of whole titanium dental implants, Bermejo et al., comparing different locations of the implant threads, found that the distribution of viable bacteria was not uniform over the dental implants; hence, macrostructure implant design might also play a role in the pattern of bacterial colonization [22, 23].

Since *in vitro* studies of biofilm formation on dental implant surfaces were mostly performed by utilizing titanium and zirconia disks [14–17, 19–21], the effect of macrostructure implant design could not be determined. The pattern of biofilm development could be different along the whole dental implant [23]; nevertheless, it is rarely investigated. Therefore, the present study is aimed at comparing titanium and zirconia implants regarding the amount of biofilm formation at different time frames using a four-species biofilm model and assessing the distribution of biofilm on different aspects of dental implants.

## 2. Methods

### 2.1. Implant Materials

The dental implants^∗^ used in this study are commercially available in the United States (Straumann® Dental Implant System, Institut Straumann AG, Basel, Switzerland). Each group consisted of nine dental implants. In the test group, sterile one-piece zirconia (Zr) dental implants with zirconia large-grit sandblasted and acid-etched (ZLA) surface with machined surface neck of 1.8 mm, made of 100% yttria-stabilized zirconia (Straumann® PURE Ceramic Implant Monotype), were used. Sterile bone-level tapered titanium (Ti) dental implants with sandblasted and acid-etched (SLA) surface, made of pure titanium grade 4 (Straumann® titanium SLA® implants), were served as control. The size of the dental implants for both groups was 3.3 mm in diameter and 8 mm in length with a thread pitch of 0.8 mm. Previous studies demonstrated that the arithmetic mean surface roughness (Sa) of the implant materials was 1.2-1.5 *μ*m for Ti implants with SLA surface [16, 24, 25] and 0.58-0.63 *μ*m for Zr implants with ZLA surface [16, 24].

### 2.2. Bacterial Strains and Culture Conditions

Standard strains from the American Type Culture Collection (ATCC; American Type Culture Collection, Manassas, VA, USA) were used. A four-species biofilm consisting of *S. oralis* ATCC 35037, *A. naeslundii* ATCC 12104, *V. dispar* ATCC 17748, and *P. gingivalis* W83 was used. Biofilms were grown in brain heart infusion (BHI) culture medium supplemented with 1% yeast extract, hemin (0.5 mg/mL), and menadione (5 mg/mL). Each bacterial species was incubated separately for 24 hours at 37°C under anaerobic conditions (80% N_2_, 10% H_2_, and 10% CO_2_). For all 4 microorganisms, each coculture started from OD_600_ = 0.1, which was diluted in the medium resulting in OD_600_ = 0.01 [26, 27], which was equivalent to ~10^5^ CFU/mL [27]. Following the establishment of the multibacterial species, each of the Ti and Zr implants was placed in the horizontal direction into sterile 24-well microtiter plate. 1 mL of the four-species bacterial suspension was added with the use of sterile pipettes. The plates, covered with the plastic lids, were incubated for 3 and 14 days at 37°C in anaerobic conditions (80% N_2_, 10% H_2_, and 10% CO_2_). The spent culture medium was replaced every 24 hours by fresh sterile culture medium.

### 2.3. Biofilm Treatments and Analysis

After 3 or 14 days of biofilm formation, dental implants containing biofilms were transferred to sterile tubes containing 5 mL of 0.89% NaCl. Biofilm suspensions were sonicated (three 10 s pulses, 7 W output). An aliquot (0.1 mL) of the homogenized suspension was serially diluted and seeded onto 5% anaerobe sheep blood agar plates (Becton, Dickinson and Company, Sparks, MD, USA). The plates were incubated at 37°C in anaerobic conditions for 7 days, and then, the CFU was calculated. Species-specific CFU was not obtained. Given the different volumes of Ti and Zr implants, biomass was measured with dry weight to normalize CFU data, which is presented in CFU/mg of biofilm. For the dry weight, 3 mL of cold 100% ethanol was added to 1 mL of biofilm suspension. The suspension was then centrifuged at 5000 × g for 10 minutes at 4°C. After discarding the supernatant and adding 1 mL of cold 100% ethanol, the sample in each tube was transferred to the preweighted microcentrifuge tubes. The microcentrifuge tubes were centrifuged at 14000 rpm for 10 minutes. Following the removal of the supernatant, the samples were dried in a desiccator for 7 days. CFU/mg was calculated after weighting the tubes with the use of a precision balance.

### 2.4. Scanning Electron Microscope (SEM)

**Figure 1:**
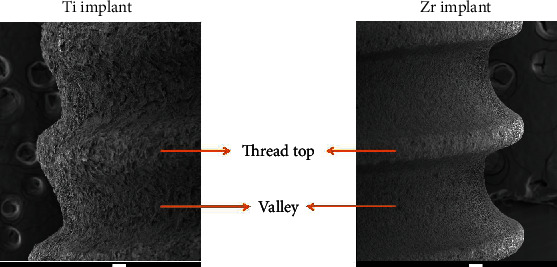
SEM images of implant body (60x; scale bar 100 *μ*M).

After 3 and 14 days of incubation, the implants were washed three times with phosphate-buffered saline (PBS) and fixed by 2.5% glutaraldehyde. A series of ethanol solutions (80%, 90%, and 100%) were used for dehydration of the implants. Following drying the specimens in a desiccator for 7 days, gold sputter coating of implants was performed. The distribution of biofilm formation on the implant surfaces, including the thread top and valley areas of the implant body (Figure 1) and the coronal portion of the implant, was analyzed with a scanning electron microscope (JEOL 7800f Field Emission, Akishima, Tokyo, Japan). The magnification was at 2300x for capturing the biofilm images and at 60x for the implant body pictures.

### 2.5. Statistical Analysis

This study methodology and statistical analysis were reviewed and approved by an independent statistician (G.E.). Two-way ANOVA was used to examine the effects of implant type and time on CFU. Log-transformed data was used to satisfy the ANOVA assumptions. A 5% significance level was used for all tests. Analyses were performed using SAS version 9.4 (SAS Institute, Inc., Cary, NC, USA).

## 3. Results

### 3.1. Quantitative Analysis by CFU/mg of Biofilm

With a total of nine implants in each group, Table 1 demonstrates the CFU/mg data for 3- and 14-day-old biofilms for the Ti and Zr groups. CFU/mg for 3-day-old biofilm on Ti implants was significantly higher than that on Zr implants (*p* < 0.001). However, for both Ti and Zr groups, CFU/mg was not significantly different for 14-day-old biofilm (*p* = 0.534). While significantly higher CFU/mg was recovered from biofilms formed on Zr implants after 14 days of biofilm development compared to that after 3 days (*p* = 0.016), the Ti groups had more viable bacteria for 3-day-old biofilm compared to 14-day-old biofilm (*p* = 0.025).

### 3.2. Qualitative Analysis by SEM

**Figure 2:**
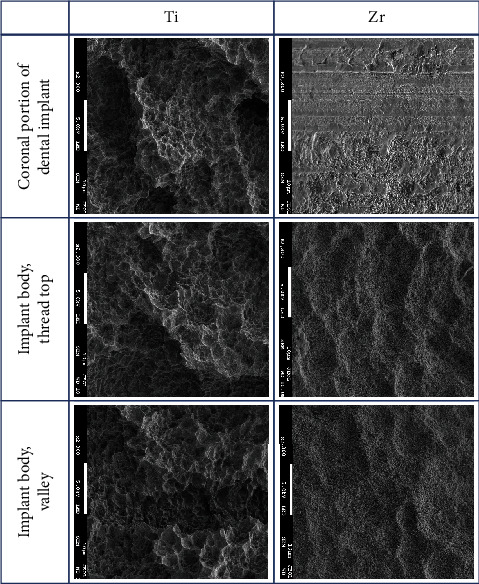
SEM images of sterile Ti and Zr implants (2300x; scale bar 10 *μ*M).

**Figure 3:**
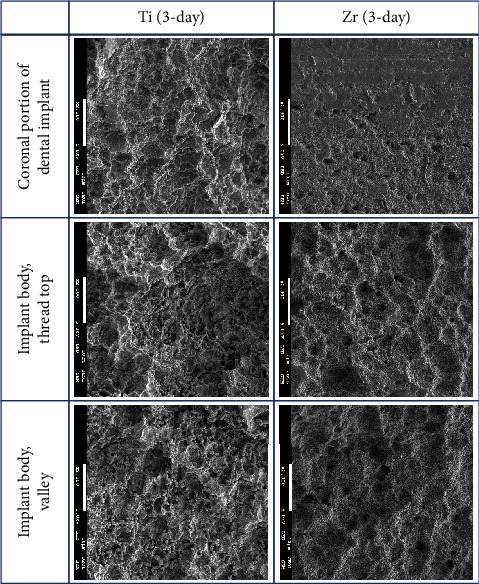
SEM images of 3-day-old biofilms on Ti and Zr implants (2300x; scale bar 10 *μ*M).

**Figure 4:**
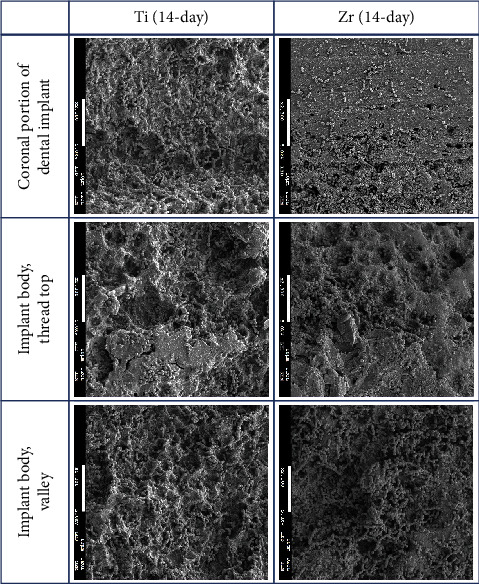
SEM images of 14-day-old biofilms on Ti and Zr implants (2300x; scale bar 10 *μ*M).

The implant body for Ti and Zr implants was rough surfaces whereas the most coronal portion of the Zr implants was smooth surface (Figure 2). Three-day-old biofilm on Zr implants was sparse while mature biofilm formation was observed for other groups (Figure 3). The 14-day-old biofilm on the smooth surface part of the Zr implants was thinner in comparison with the implant body, where the biofilms were thick and clustered. Visually, it appeared that less biofilm formed in the valley compared to the thread top for 3-day-old biofilm on Zr implants. In contrast, as the biofilm developed on the implant surfaces, the differences between the valley and the thread top became indistinguishable (Figure 4).

## 4. Discussion

The present *in vitro* study, with the use of peri-implantitis-related multispecies biofilm model, is aimed at comparing the biofilm formation on Zr versus Ti implants and investigating the effect of incubation time on the biofilm development. To the best of the authors' knowledge, few studies investigating bacterial attachment on implant materials utilized commercially available dental implants.

Similar to gingivitis and periodontitis, bacteria play a critical role in the development and/or progression of peri-implant diseases [28]. Thus, a material which could decrease bacterial attachment would be ideal from the view of preventing/slowing the inflammatory response around peri-implant tissues. The study by Scarano et al. showed that, by having the subjects wear removable acrylic device for 24 hours, lower bacterial adhesion was observed on zirconium oxide surface in comparison with that on pure titanium disks though the surface roughness for both materials was similar [15]. An animal study revealed that the progression of bone loss around Zr implants was slower than that around Ti implants during the phase of active progression, a period when cotton ligatures were placed around implant shoulders to induce peri-implantitis [12]. The results from the current study, in which Zr implants had less biofilm formation at an earlier time point when compared to Ti implants, may, at least partly, explain their findings. On the other hand, the number of bacteria was comparable between Ti and Zr implants after the biofilms developed for a longer time. This seemed to be corresponding to the results of the *in vivo* study, in which no significant difference was observed for the changes of crestal bone level around Ti and Zr implants at a later time point, although the authors of the study speculated that implant materials might be the more important factor in terms of peri-implant bone loss during active progression period whereas implant surface topography played a more crucial role during the spontaneous progression period [12].

Surface topography has been recognized as one of the critical factors affecting bacterial attachment [29, 30]. Due to the design of the Zr implants used in the present investigation, biofilms formed on the smooth and rough surfaces could be observed simultaneously. As the biofilms became more mature, bacterial cells tended to be clustered on the rough surfaces while the biofilm cells seemed to be scattered on the smooth surfaces, thereby rendering the visual impression of more bacteria growing on the rough surfaces. In line with a study comparing biofilm formation on smooth- and rough-surfaced Ti and Zr disks, the biofilm thickness was statistically higher on rough-surfaced Zr groups than that on smooth-surfaced Zr disks in both human plaque biofilm and an *in vitro* three-species biofilm model despite the fact that the biofilm mass between smooth- and rough-surfaced Zr disks was comparable [16]. In contrast, Sanchez et al. found that six-species biofilms exhibited a honeycomb appearance on rough-surfaced Ti disks while a cobwebbed appearance was present on the zirconia groups, which had a rough microsurface, after 72 hours of incubation [17].

Quorum sensing (QS), a process in which bacteria communicate and coordinate with one another through small signaling molecules, autoinducers, is critical for the development of biofilm [31, 32]. Autoinducer 2 (AI-2) can be produced by Gram-positive and Gram-negative bacteria. Mutualism between *S. oralis* and *A. naeslundii* seems to be dependent on AI-2 [33]. On the other hand, *P. gingivalis* produces *N*-acyl homoserine lactones (AHLs), which could be of vital importance to oral biofilm development [34]. The spatial distribution of bacterial cells may play a significant role in determining the concentration of autoinducer [31]. The induction process, in which the autoinducer reaches a threshold value and thereby regulating gene expression, occurs earlier when the cells are clustered, exhibiting an inhomogeneous distribution, than those that are more evenly distributed [32]. Hence, it is possible that, in addition to surface topography, the different patterns of biofilms formed on the smooth and rough surfaces were also affected by QS.

The biofilms were not uniformly distributed over the whole dental implants as more bacteria were found at the peak and lateral surfaces of the implant threads compared to the area between threads, as demonstrated in studies by Bermejo et al. [22, 23] This is in agreement with the present study in part. Early formed biofilms on Zr implants appeared to accumulate more on the implant thread top regions than the valley areas. Yet, the biofilm formation on Zr and Ti implants seemed to be comparable as the biofilm developed more maturely based on quantitative assessment and visual analysis. It has been suggested that decontamination of implant surfaces is critical to the treatment of peri-implant diseases [28]. Various instruments for decontamination of dental implants were examined in an *in vitro* study. Among the tested instruments, the valley areas could only be accessed by titanium brush and a glycine-based air-powder abrasive device [35]. Treatment outcomes of peri-implantitis varied. While >70% of implant survival rate was reported at 7-year follow-up, the success of the treatment, still, appeared to be not very promising as the estimated cumulative success was calculated as 34-57% for 3 years and 7-41% for 7 years [36]. Given the mature biofilms present at peri-implantitis sites, the inability to decontaminate some areas of implant surfaces may account for the current challenge in the management of peri-implant diseases.

The multispecies biofilm model employed in this study is aimed at mimicking *in vivo* peri-implant biofilms and was based on a validated model [26], consisting of initial, early colonizers (*S. oralis*, *A. naeslundii*, and *V. dispar*) and *P. gingivalis*, which has been associated with peri-implant diseases; one advantage of this model is its reproducibility [26]. It was somewhat unexpected that the quantitative assessment revealed that there are more viable bacterial cells attached on the titanium implants in 3 days compared to 14 days. Nonetheless, this might not be biologically significant in view of the differences in absolute CFU/mg value between 3 and 14 days.

Utilizing two-piece Ti implants and one-piece Zr implants is one of the limitations of the present study. Burgers et al. demonstrated that rough Ti surfaces had significantly higher initial bacterial adhesion compared to machined surface Ti specimens [20]. This implies that using one-piece Ti implants might render a lower CFU, and therefore, the level of statistical significance could be affected if one-piece Ti implants were used.

In this investigation, the use of commercially available dental implants could be considered as more clinically relevant. However, the nature of *in vitro* models is the limitation of this study in so much as, in addition to the complex microbial composition and interaction *in vivo*, the effects of host response and peri-implant environment could not be evaluated. The inflammatory responses of peri-implant soft tissues appear to be more pronounced around titanium surfaces than zirconia surfaces [37]. Additionally, titanium particles and/or ions released from implant surfaces might pose a negative impact on peri-implant tissues [38]. Therefore, further research investigating the interaction among peri-implant tissues, microbes, and implant materials remains to be explored.

## 5. Conclusion

In conclusion, while early formed biofilms show greater accumulation on titanium implants compared to zirconia implants, older biofilms between the two groups are comparable. The macrostructure of dental implants may affect the distribution of biofilms during early biofilm development though its influence seems to be diminished as the biofilms become mature.

## Figures and Tables

**Table 1:** Mean and standard deviation of Log_10_ CFU/mg on 3-day-old and 14-day-old-biofilms. Two-way ANOVA was used to examine the effects of implant type and time on CFU (*α* = 0.05).

	Mean ± SD (CFU/mg)		*p* value
	Ti	Zr	
3-day-old biofilm	3.67 ± 0.94	2.17 ± 0.57	<0.001
14-day-old biofilm	2.86 ± 0.79	3.08 ± 0.65	0.534
*p* value	0.025	0.016	

## Data Availability

The raw data used to support the findings of this study are available from the corresponding author upon request.

## Abbreviations

SEM: Scanning electron microscopy
CFU: Colony forming unit
Ti: Titanium
Zr: Zirconia
SLA: Sandblasted and acid-etched
ZLA: Zirconia large-grit sandblasted and acid-etched.
